# The Last Sentiments of Suicides

**Published:** 1851-04-01

**Authors:** A. Brierre de Boismont


### THE LAST SENTIMENTS OF SUICIDES,

BY

DR. A. BRIERRE DE BOISMONT.

*(Translated from the Author's MSS.)*

In the history of suicides there is a very sad but painfully interesting chapter, the analysis of the sentiments expressed by these voluntary victims in their last moments. To give to this new and instructive subject the study which its importance deserves, it was necessary to consult a number of documents sufficient to warrant some confidence in the conclusions arrived at. Among the 4595 facts which constitute the basis of this memoir, we found 1328 letters, notes, and writings of different kinds,* in which the diversified secret workings of the human soul are revealed. Upon subtracting the number of illiterate persons, who could not write, from the total number of suicides, we find that very few are willing to quit this life without leaving behind them some kind of souvenir—some

* Of these writings—69 were in pencil; 10 in chalk, on the walls; 8 in their pocket-books; 17 on the doors, windows, shutters, floor, or wood-work of the room; 2 on parchment; 2 on the table; 3 on paper attached to the clothes; 19 were written in a firm legible hand, which might serve for a copy; 63 out of the total number contained some testamentary bequest. The number of writings found during the ten years, 1834 to 1843, inclusive, was thus divided: 128, 137, 141, 156, 132, 149, 138, 100, 114, 133.

record of their sufferings and misfortunes; of their blighted hopes; of their withered and wasted affections. The desire of being still remembered, of leaving some memento of their passage through the world, seems the predominant motive of the greater number. And is not this instinctive dread of oblivion—this general and invincible repugnance which all men entertain to the idea of a total and final death—a strong argument in proof of the immortality of the soul? A second fact which we arrive at from a philosophic analysis of these documents, is, that when man is liberated from the artificial trammels of society, and ceases to be governed by the paltry passions of the hour, then the good and generous sentiments of his nature prevail. We do not mean to assert that it is always so—for these documents prove the existence of dispositions unmixedly perverse,—still we state the truth in saying, that the good sentiments greatly exceed the bad.

M. Guerry, in his "*Essai de Statistique Morale de la France,*" has traced in a few lines, a summary of the sentiments commonly expressed by suicides, drawn from 100 letters. We will now give a table with the results of our examination of 1328 autograph documents, premising, that as many of these contain more than one sentiment, they are entered under two or more heads, so that the sum total is 1557; 1204 men, 353 women, instead of 1328; 1052 men and 276 women.

*A general Table of the Sentiments expressed by Suicides in their last Writings, arranged numerically.*

| Males. | Females. | |
|---|---|---|
| 217 | 87 | Reproaches, complaints, declamations, reflections on the causes of their death. |
| 218 | 60 | Farewell to relations, friends, and acquaintances; to the world. |
| 192 | 45 | Declamatory complaints against life: it is a burden. |
| 56 | 11 | Instructions for their funeral. |
| 48 | 9 | Say that they are of sound mind, and accuse no one of their death. |
| 43 | 12 | Say that their mind is confused. |
| 44 | 4 | Avowal of a crime, a criminal attachment, a bad action. |
| 36 | 9 | Pray to obtain pardon for their suicide; wish to be recognised. |
| 30 | 13 | Solicitude for future welfare of parents, children, relatives, &c. |
| 21 | 15 | Confidence in the mercy of God. |
| 25 | 6 | Benevolent wishes. |
| 26 | 5 | False motives. |
| 28 | 1 | Materialism. |
| 12 | 12 | Instructions as to the manner of their burial. |
| 20 | 2 | Regrets of life. |
| 18 | 4 | Belief in a future existence. |
| 13 | 5 | Die honourably. |
| 5 | 11 | Regret at separating from a beloved person. |
| 13 | 2 | Desire to expiate a fault. |
| 9 | 6 | Pray forgiveness for errors of their past life. |
| 9 | 2 | Pray their friends to give a tear to their memory. |
| 10 | 1 | Request the prayers of the Church. |
| 10 | 1 | Wish to be carried at once to the cemetery. |
| 9 | 2 | Futile motives. |
| 9 | 0 | Horror at the act they are about to commit. |
| 0 | 9 | Despair at having yielded to seduction. |
| 8 | 1 | Hope that publicity will not be given to their act. |
| 7 | 2 | Depraved and dissolute ideas. |
| 7 | 1 | Agony of mind. |
| 5 | 3 | Belief in fatalism. |
| 6 | 2 | Indifference of what is thought of their action. |
| 5 | 3 | Wish to have a ring, or other souvenir, buried with them. |
| 7 | 1 | Prayer that the manner of their death may be concealed from their children. |

| Males. | Females. | |
|---|---|---|
| 6 | 1 | Desire to be buried as paupers. |
| 5 | 1 | Commend their souls to God. |
| 5 | 0 | Express long hesitation. |
| 3 | 1 | Consider themselves useless, an encumbrance on the earth. |
| 3 | 0 | Dread of the suffering they are about to undergo. |
| 3 | 0 | Fear of wanting courage. |
| 2 | 1 | Bequeath a lock of their hair. |
| 3 | 0 | Sketch their vanished hopes. |
| 1 | 1 | Regret inability to show their gratitude. |
| 2 | 1 | Apprehension of being exposed at "La Morgue." |
| 2 | 0 | Speculation on the lot of their corpse. |
| 1 | 0 | Invitation to publish the letter in the newspapers. |
| 1 | 0 | Insults addressed to the clergy. |
| 1 | 0 | Incertitude concerning future destiny. |
| 1204 | 353—1557 | |

To facilitate the analysis of these sentiments, we will divide them, after their nature, into three classes, at the same time observing, that this division is by no means rigorous. In the first we will arrange the sentiments of benevolence, repentance, religion, honour, tenderness, friendship, gratitude, &c., and unite them under denomination of *good sentiments*. In the second we will place the sentiments of reproach, resentment, vengeance, complaint, imprecation, disgust of life, materialism, irreligion, debauchery, hypocrisy, &c. &c., calling them *bad sentiments*. Afterwards, we will group in a third class those sentiments which do not strictly and exclusively belong to either of the foregoing classes, though partaking in a greater or lesser degree the nature of both, and we will call these the *mixed sentiments*.

### I. Good Sentiments.

This section comprises the analysis of nineteen varieties of sentiments, which may be further divided into five sub-sections. The proportion of cases in this class is 626 (474 males, 152 females).

1st Sub-section.—*Farewell to relatives, friends and aquaintances, to the world, announcement of death, last will, recommendations, wishes.*

To bid a last adieu to the world they are about to quit, to give some token of affection or friendship, to reveal their troubles and regrets to the persons they have known, such is the sentiment the most commonly expressed by suicides, in their writings. The number of these amounts to 278 (218 men and 60 women). This desire is sometimes so strong, that, wanting friends or acquaintances, they address their adieus to inanimate nature, exclaiming, with Gilbert,

"Farewell, beloved fields and sweet green meads,"

thus manifesting that instinctive love of our first mother which is rarely altogether extinguished in the bosoms even of those whom cruelty, injustice, or fancied wrong, have alienated and separated form their kind.

In the way of expressing this sentiment, there is a veritable hierarchy; thus, in the first place, come the adieus to their own family, beginning with those addressed to a husband or a wife. Friends and companions are not forgotten in that fatal hour, especially by men, who form nineteen-twentieths of the amount, which goes to confirm the remark of the satirist, that women never have a friend of their own sex.

Adieus to lovers and mistresses hold the fourth place; but here the proportion of the male sex, which in the preceding list has been very superior, falls to a level with the female, thus tending to establish Madame de

Staël's opinion, that love is a mere episode in man's life, but the very history of the life of woman. Adieus to the world at large come from men only. Lastly, come adieus of servants to their masters; they are but few in number. Suicides do not confine themselves to bidding adieu; they also announce that they destroy themselves, very commonly, without stating the motive: 202 individuals (166 men and 36 women) are comprised in this category. The expressions usually employed are these: "I destroy myself by my own free act—when this letter is received I shall have ceased to live—I alone am the author of my death—as well to-day as to-morrow—it is all over; my last thought was of thee—they will hear of me to-morrow—it is here that I must die—I leave for the next world—it is I myself—no one will see me more—I am about to die—accuse me alone, and trouble no one else—I cannot reveal the reason of my death to any living soul—I am about to do that which I ought to have done long ago—it is necessary that I die—I profit by the absence of my companion to terminate my existence—my death approaches—I blow out my brains—my resolution is fixed—it is two o'clock in the morning, and I am dying; as suffocation comes too slowly, I burn all my essences—my friends, it is midnight, the fire is lit, you repose, to be able to resume your work, but I wish never to rise again—if this fails, water shall do it—to-day I bury myself in water—I threw myself in," &c. &c.

Thirty-nine individuals (23 males and 16 females), in bidding farewell, also explain the motives which led them to commit the act; these motives are those indicated in the chapter of causes. As this fact will reappear in the analysis of all the sentiments expressed by suicides in dying, we proceed to give a general table of causes drawn up from an examination of the writings found and preserved in the official reports.

*Summary of Causes indicated in* 1328 *writings.*

| | |
|---|---|
| Trouble, real or imaginary | 176 |
| Love | 154 |
| Weariness of life | 141 |
| Domestic troubles | 101 |
| Debts, ruin | 96 |
| Illness | 65 |
| Poverty, misery | 55 |
| Insanity | 40 |
| Bad actions, remorse | 32 |
| False motives | 31 |
| Misconduct | 19 |
| Gambling | 13 |
| Pride, vanity | 10 |
| Intemperance | 9 |
| Unknown causes | 386* |
| | 1328 |

The general impression which this list conveys is, that moral suffering has a very different effect from physical suffering—a point which we have signalized in previous works.†

A certain number of suicides, 43 (36 men, 7 women), in their farewell

* These 386 writings, although they do not furnish any information on the causes, have revealed to us many curious and important peculiarities of the character, principles, &c., of suicides.

† "De l'influence de la civilisation sur la developpement de la folie," Annal. d' Hygiène, tom. xxi. p. 241–295. 1839. "Des maladies mentales," Bibliothèque du Médecin Practicien, tom. ix. p. 366.

letters, make known their wishes, express their last requests, and offer recommendations. We may specify these sentiments in the following manner:—Expression of thanks and gratitude towards persons who have done them service, or who have sympathized with their troubles—desire or hope that their death will make their family more happy—wishes that their friends may lead a happier life—prayer to banish their memory—recommendation to employ all possible precautions in communicating their death to their family—regret at having nothing to bequeath—direction to send their effects to their relatives—hope that pity will be shown to those whom they abandon—exhortation to industry and good conduct—distribution of what they die possessed of. The final recommendations may be thus classed:—To forward their effects to relatives or persons to whom they belong—to pay their debts—to destroy all their papers. "My dear son," says one, "burn my books without opening them: it is my last request." Others desire crucial incisions to be made in the soles of their feet. One man mentions, that having fallen into a lethargy at the age of seven years, he was on the point of being buried alive. Many direct the windows to be opened as soon as the room is entered,—to carry their remains to their family—to come and see them before all is over—to make no inquiries about them, &c. &c.

2nd Sub-section.—*Avowal of a crime, of a bad action, of a criminal passion; desire of expiation; prayer for forgiveness; declaration of honour, &c.*

The voice of conscience can never be wholly stifled. Even if the recognition of a crime has escaped human justice, an inner witness ceases not to warn the criminal. In insanity, an hallucination is often the personification of remorse.

In 48 cases (44 men and 4 women) our notes show that the memory of an evil deed was the cause of suicide. The motives of these 48 voluntary deaths offer themselves under three principal heads:—Crimes (18); bad actions (15); and criminal passions (15).

Sometimes the crimes are avowed, at others concealed. "I die," says one man, "of remorse and despair, to avoid the punishment of a crime that I alone know. I was unwilling to dishonour my family. I have this night seen the woman I adored die in my arms, self-poisoned because she would not survive me." Another expresses himself in these terms: "When you receive this letter I shall no longer exist. I have committed a crime which would have condemned me to the galleys, and I have now no other resource than to blow out my brains. Adieu! dear parents; I feel my hand tremble, and my ideas become confused, so it is time for me to render an account of my deeds on high. All that I ask of you is, not to afflict yourselves for me, for I am altogether unworthy of your regret." A third says: "To live dishonoured in your eyes, or to quit a life endurable only with your affection, there can be no hesitation in my choice; and I regret this decision only on account of the sorrow it may cause you. Forgive me, and do not curse the memory of one who was to you a well-beloved son; to thee, my good L——, a dearest brother. I give you, my darling sister, my ring, which you will find; speak kindly of me sometimes to your little girl, whom I loved as fondly as her father. I have destroyed myself by my own act. I beseech the persons who find me to inform my family with all possible consideration." Many letters contain some such reflections as the following: "I have found here only shame and dishonour, so leave it."—"I am more weak than guilty."—"I have done justice on myself for my crimes."

Bad actions, and faults of various kinds, are, for timid souls, and those brought up with a sense of duty, a constant source of self-reproach. In a letter found by a dead body, we read these words: "Induced, on Wednesday, by a man I will not make known, but on whom my end will make a terrible impression, [perhaps his father!] I spent with him a sum of money which was not mine, and which I cannot repay you. I have punished myself for it."—"A portrait," writes a lady, "found by my husband after our marriage, by revealing a fault I thought concealed for ever, destroyed my position, and shattered all my prospects. To avoid his terrible reproaches, the hate of my family, and the scandal of a divorce, I prefer to kill myself. A moment's suffering cannot be weighed against a whole life of torment and misfortune." A young man leaves a letter for one of his friends, in which he announces his regret at dying at 28 years of age; but that he cannot live any longer with honour, because his folly had led him into very grave faults, and made him the sorrow of his family. A man contracts a shameful disease, and infects his wife: he says to her—"My darling, you do not make me a single reproach, but those which I address myself are so violent that they will drive me mad. Forget a wretch unworthy of thee, and who ought to have been the very last man to commit such a crime."

The regrets which the passions leave behind are frequently so poignant that death alone can terminate them. A gamester announces his ruin to his family, and felicitates himself that he had previously divided a portion of his fortune among his children, who, without that, would have been left utterly destitute. He finishes his letter by a doggrel rhyme, to the effect that death is the best cure for hunger. "I am so completely subjugated by my incorrigible propensity," writes a man to his family, "I have given you such grave grounds for anger, that my only resource is to die."—An artisan makes this confession: "Being unable to conquer my taste for drinking and debauch, I prefer destroying myself before I am reduced to beggary." The majority express their pain at not being able to correct their bad habits, and deplore the excesses into which they have been led by them.

*Expiation: the desire to expiate a fault.*—To the confession of a fault there often follows the desire to expiate it. Fifteen persons (thirteen men and two women) show this in their last writings. Here, it is a husband who writes to his wife: "Seeing myself engulphed in a life of disorder and debauch, without the strength to extricate myself from it, in spite of the reproaches which I make myself daily, I prefer offering up my existence as a kind of expiation for my bad conduct, rather than any longer run the risk of losing the affections of my friends, and incurring fresh dishonour; but I get on too quickly, so I stop myself, and trust that God will pardon my act in consideration of the motive." There, it is a wife who criminates herself to her husband, saying that death alone can expiate her fault. She recalls to him their former happiness, and the pleasant time they had passed together, protesting that she had never ceased to love him, that circumstances alone had overcome her sense of duty, and that she herself did justice on her own frailty. Another time it is a father who has dissipated the entire inheritance of himself and children, leaving them in misery; on the table beside his body are found several letters from his wife, conjuring him, in the most pathetic terms to change his conduct, and not abandon his children, but to come to their assistance, as she is unable any longer herself to support and educate them.

Many state that they kill themselves in expiation of a fault or crime which they will not discover. Others, that they punish themselves for an abuse of confidence, misconduct, adultery, or darker crimes; for having brought disgrace and misfortune on their family and friends. One of these individuals thus expresses himself: "I have never loved anything but gold; my hasty temperament has driven me to commit very reprehensible actions, I am tempted to commit still worse; I might some day have to ascend the scaffold; death will cut short all these follies, and save me from that catastrophe."

*Avowal of faults; prayer to be forgiven.*—Regret for past offences is at the bottom of the heart of the greatest number, but pride retards and prevents the avowal, so that ruin and death are often preferred. Fifteen suicides (nine men, six women) acknowledge the wrong they have committed, and pray to be forgiven. A young girl writes to her parents: "Forget all my misdeeds, but do not curse me, too guilty though I be; your unfortunate child has sunk beneath her shame—oh, forgive me! I conjure you on my knees, in the face of death, do not give me your malediction. Pray for me." On the table of a student was found a letter from his father, dated two years previously, in which he points out to his son the sad career that he is about to pursue, the ills that await him, his vain regrets, and the fate that will befal him. At the bottom of the letter the son had written these words—"You were right in every particular, I trust that my death will disarm your just anger." Several females confess their infidelity to their husbands or lovers, and implore their forgiveness. Some few men make the same avowal, saying that their death is a just punishment for their misconduct.

In opposition to the foregoing, some destroy themselves because they cannot support the idea of being suspected, accused, calumniated, &c.; they are the victims of an exaggerated sense of honour.

*Die men of honour; women of character.*—"Monarchies live by honour, republics by virtue," says Montesquieu. In France, the first of these sentiments has caused torrents of blood to flow. During many ages, some millions of men risked their lives in single combat, frequently in spite of severe laws, at the least attack upon their honour. It is the exaggeration of this sentiment which drives a great number of unfortunates to destroy themselves. In eighteen instances (thirteen men and five women) we have found this stated in the letters left by the suicides. The antique probity of commerce, formerly so general, which made bankruptcy an irreparable misfortune, was the motive which determined six merchants or tradesmen to put an end to their existence. One of them, arrived at an advanced age, declares that the impossibility of meeting his engagements is the sole cause of his fatal resolution. "I have done everything to struggle against the torrent which has overwhelmed me; all my efforts have proved unavailing. I have 200 francs in the drawer of my secretaire, which will serve to defray the expenses of my burial, which I wish performed as economically as possible. I pray my creditors to forgive me if they have lost anything by me; I can assure them it has not been from any fault of mine, for I cannot reproach myself with the least unnecessary expense. Midnight; one hour before my death." This letter is written in a firm hand, differing in no respect from his writing in his ledger. To the cause above indicated, may be added the discouragement natural to old age, which affords no possibility of beginning life anew. Another merchant writes to his wife: "Thirty years of irreproachable probity will not allow me to endure a protest. After a time, perhaps, all might be repaired, but the remembrance of this bankruptcy would kill me by slow degrees. I prefer finishing at once. I have taken precautions that this event shall give you as little trouble as possible."

A certain number declare that they die men of honour, without giving any further explanation. "My troubles are beyond my strength," writes one of these; "I would rather die than live dishonoured. Bury me with the rites of the church, and tell my father to remember the 3rd of January,

18—." Another announces that he cannot survive the infamous calumnies which have tarnished his reputation—the dearest thing to him on earth. His conscience is pure, and he dies forgiving his calumniators.

The motives alleged by women relate almost exclusively to their morals. "I love a young man," says one of them in her letter, "but I have not yielded to him, which may be easily verified; it is this calumny which kills me." Another writes, "I have made a thousand attempts to procure work, but I have found only hearts of stone, or *debauchés*, whose infamous proposals I refused to listen to." A young girl, strikingly beautiful, states that she has exhausted all her resources, and left all her effects in pawn. She adds, "Had I chosen, I might have had a shop, richly stocked, but I would rather die chaste than live disreputably."

Business matters have much less effect on women than on men.

3rd Sub-section.—*Demand pardon for their suicide; solicitude for beloved persons; regrets at leaving them; prayers to be forgotten; to come to recognise them.*—The man who has resolved to finish his career still thinks of those he leaves behind, and asks their pardon for the grief and trouble which he is about to cause them. Forty-five letters (thirty-six men and nine women) prove their solicitude on this point. They are mostly addressed to relations, some to friends, and even to strangers; they express the grief of the writer at having to quit them, alleging some imperious motive, some despair, which leaves them not a moment of repose. "My dearest wife," writes a broker, "forgive me the suffering I am about to cause you, and which will be augmented by the discovery of the deranged condition of my affairs: and you also, my mother, pardon me this blow, so heavy at your age, you whom I loved so dearly, and who had such just grounds to rely upon your children; my evil destiny has prevailed." A wife confesses to her husband that her resolve had been made for some time, because it was impossible for her to exist apart from some other man whom she adored; her letter is written in a firm hand, and she executes her project calmly and deliberately. The celebrated painter, G——, left these words, in pencil, in his note-book: "Mr. B—— will entreat my dear wife. I have now nothing more to say but good-bye, dear wife."

Many of these unfortunate persons, after having besought forgiveness for their deed, request that their remains may be identified, and the last services to the dead accorded them. "One more favour," writes a man; "you will proceed immediately to the Champ de Mars, to identify my corpse; for when you arrive there, I shall exist no longer."

*Solicitude for the future welfare of children or relations.*—The domestic affections are not wanting in suicides, and their writings often disclose all the agony of their souls. The number of letters in which this sentiment is expressed amounts to 43 (30 men, 13 women); and it will be observed that the relative proportion of females here becomes more considerable. Anxiety about children is the predominating sentiment, and is exhibited in 40 cases (25 men, 15 women). These poor creatures recommend them to their relations, to their friends, to charitable persons; they lay down rules for their conduct,—they give them their blessing, and manifest the most poignant grief at being forced to part from them. A man beseeches his wife not to marry again until his son has passed through the conscription, and his daughter been confirmed, placed apprentice in a good establishment, and proved to be steady; he says that he has never been happy in this world, and so hopes for a better. A father writes a very affectionate letter to his children, informing them that for their sakes he is unwilling to marry again, but fearing to be led away in spite of himself, he prefers dying. Life is full of these irresistible impulses. How often do we see the unfortunate victims of some organic disease abandon themselves to pleasures which are so many mortal strokes to them, in spite of the repeated energetic protests of their reason: they are aware of their danger; they promise themselves to resist, but they fail again and again in their resolution, till they sink to rise no more. What, then, is the use of reason? Who profits by it? Some few men, born without passions; the infinitely small number who have learnt to subjugate them; some remnant of those who have exhausted them in youth, and whose ardour is chilled by age.

Solicitude for other relatives is manifested much less frequently than for children, and is more commonly shown for wives or mistresses than for parents: it is characterized by regret for the grief their death will cause, and the pecuniary distress it may occasion.

*Good wishes and tender sentiments to friends, benefactors, acquaintances, enemies; regret at leaving no means of showing their gratitude; forgiveness.*—If, on the one hand, many men go down into the grave with all their evil passions, resentments, dislikes, and hate uncancelled and unappeased, yet, on the other, there are many who, guided by a better spirit, forgive the wrongs inflicted on them, and forget the insults or injuries they have endured. Indeed, it seems incredible that any one, in the least degree influenced by religion or morality, could resolve to present himself before his Maker with a heart full of gall and rancour. The number of those in whom these better sentiments were manifested is thirty-three (twenty-six men, seven women). Here are some fragments of their letters: "If I have injured any one, let me be forgiven; in killing myself, all should be forgotten. One last thought of my son and daughter. I die in full possession of my faculties. Let my ashes be respected. I have suffered keenly without complaining. The only person I have never injured has rendered life odious to me, but I forgive. I could have avenged myself; I prefer forgetting all. I am not yet thirty, and I die. The passage from life to eternity is a mere trifle."—"You will oblige me by informing my family of this sad affair, and, at the same time, assure them that I bore no resentment for what had taken place between us during past years; I attribute all my misfortunes to my marriage, and to a supreme and inexorable fatality."—"Since all abandon me, I abandon myself; may God render as much good to my persecutors as they have wrought me evil."—"C——, when you get this, I shall be no longer alive. I regret that thou shouldst be one of the chief causes of my death, nevertheless, my last thought is of thee. Let me reiterate once again the advice I have so often given thee, and which I repeated only yesterday, work; work, if thou wishest to avoid falling into want, and art willing to free thyself from the infamous yoke thou now endurest."

Most of the other letters are from married couples, who reciprocally forgive each other's death; from persons who thank their friends or benefactors, or address a few words of reconciliation and forgiveness to their enemies.

Gratitude is at the bottom of the human heart; but, unfortunately, the conflicting passions and interests of life too often choke it, and stifle its development. Nevertheless, we have two documents in which this sentiment is strongly expressed:—"Adieu, dear parents, and you, my excellent masters," writes a female domestic. "Why did I ever leave you? After so much kindness from you! I know that I should have to try an infinity of places before I could find another like yours; so I prefer to die."—"My dear friend," says a young man, "by your devoted conduct you have retarded my death for more than a year. I thank you for the services you have rendered me. I wished not to quit the world without giving you some mark of my gratitude. I spoke of your affairs to some one under seal of secrecy. I was desirous of doing something for you, but fate willed it otherwise."

*Regret at separation.*—Time assuages every pang, and calms all our sorrows; but, in young souls, full of impatience and vivacity, the first impression of grief is often so vivid and absorbing as to prove dangerous to life. In sixteen letters, which announce a separation as the cause of suicide, eleven belong to the female sex; in fact, the necessity of quitting the man she loves, is, to a woman, the most terrible of trials. From out of this class of documents we will select the three following:—"I perish, still loving thee, my dear friend; I am innocent; rest assured that my heart has never changed; for thee I reserved that flower which God has given me." —"The harshness of my husband has hindered my making any revelation to him. I give all I can dispose of to my brother, that he may avoid my example, and that he may be able to marry her he loves."—"Sir, I am pregnant, and the child I carry is not yours: the father is a young man whom I adored, who suffocated himself three days since by reason of the reproaches of his family. As life without him is insupportable, and despair will drive me mad, I put an end to my agony." The lover, in the last case, was a law student, at Paris for three years without doing anything, whose father, finding how his son had deceived him, had ordered him to return home immediately, or else shift for himself.

Occasionally the suicide is determined, not by the death of the beloved person, but by the inevitable necessity of a separation. Women sometimes kill themselves for grief at the loss of their parents or children. One says that she cannot survive the death of her son, and desires to be buried in the same place with him.

The same motives urge men to destroy themselves, but much more rarely than women, for their number amounts to five only. In a case of double suicide, the young man announces, in his letter, that he cannot marry his mistress, and that she, about to become a mother, and fearing to be turned out of her home, and cursed by her parents, prefers death to disgrace. "I love her too well to survive her; so I follow her, to share her grave."

*Prayers to their friends to give a tear to their memory; to preserve a lock of their hair, &c.; to console those dear to them.*—There is nothing more natural than the desire to be wept for at our death by those to whom we have been attached while living; it is a consolation and a proof that we had some good quality. The following are fragments of fourteen letters (eleven men, three women) expressive of this sentiment. "My dear Eugénie, may God protect you, and may you yet find the happiness I could not procure you. Pardon me all I have made you suffer, and grant a tear to my memory. Let me be interred beside your father; I hope that your excellent qualities will be more fully appreciated. Return to your family."—"From the summit of these towers (those of Notre Dame) which I visited some days since, in company with L——, I have just now precipitated myself. Weep for me, weep for your brother, a victim of the blackest ingratitude. No doubt you will wish to see this spot moistened with my blood. As for those who have done me so much ill, I have thought of killing them, but let the wretches live, sooner or later they will receive their reward." Our nature revolts at the idea of being entirely forgotten after our death, and we seek to recall our memory by posthumous gifts. Even the suicide shows this sentiment, and distributes his memorials. "My friend, keep this bracelet in memory of me, and place a garland on the tomb of our child; such is the last request of her who loved you more than all beside."—"Please to forward my portrait to my mistress."—"I give my ring to L——; she will find it in my waistcoat pocket."

*Request that their suicide may not be made public; wish to conceal their death, and name.*—There are some men who destroy themselves through vanity, and, consequently, aim at giving to their death all possible publicity. This sentiment is often manifested by notorious criminals, who seek to finish their guilty career with *éclat.* On the other hand, there are some who expressly desire that nothing may be said about them, and no notice taken of them. We find this sentiment contained in nine letters, (eight men, one woman), variously expressed as follows: "To him who finds me I bequeath my gratitude, if he can withdraw my remains from public curiosity."—"I hope that no one will know neither my suicide nor the abode of my parents; thanks to the precautions that I have taken. The cause of my death is a secret between God and me."—"I beseech the commissary not to allow my name to appear in the papers, for the sake of my family." The recommendation to avoid publicity and the insertion of the name in the newspapers is the general sentiment in this class of suicides, and commonly springs from a wish to spare the feelings of surviving relatives or friends. In many letters the authors evince a desire to escape the idle curiosity of the public, and not to afford any satisfaction to their enemies.

*Prayer to conceal the manner of their death from their children or their parents.*—The instinct of paternal affection survives the approach of dissolution, and exhibits itself in many different ways. In the letters we have before us it is shown in eight cases (seven men and one woman). Thus, one requests his friends to spread a report that he has perished by an accident; another desires them to write and inform his family that he had been crushed by a carriage, and died in the hospital with the consolations of religion. Almost all beseech those who discover their suicide to conceal it from their children and family.

4th Sub-section.—*Religious feeling; confidence in God's mercy.*—France is the country which has produced the most admirable religious works, yet it is the country in which the practice of religion is the least observed. This anomaly is due to predominance of the imagination over the judgment, which impresses a peculiar type on our national character, and is the foundation both of our glory and our shame. In the face of death the religious sentiment often declares itself with force. Thirty-six letters or notes (twenty-one men, fifteen women) attest that these unhappy creatures at the point of death still hoped in the Divine mercy. The comparative preponderance of females over males which we noticed in the case of the domestic affections, is here even more strongly marked, and will generally be found to accompany the more sentimental ideas. From among the notes relating to the subject under consideration we may cite the following: "I kill myself to escape from a life of debauchery, of sensual indulgence, and disgrace, and to avoid losing the good will of my parents. I hope in God's pity, and trust that, in consideration of the motive of my sacrifice, he will make me happier in another world."—"I suffer too much, mother, and cannot live any longer; I must quit this earth. Pray God to forgive me, and take pity on me in another world." Many merely write that they pray God to forgive them their death, and that they have confidence in His goodness. Some few women were found with the emblems of religion about them; one had drawn a cross with charcoal on the wall, and had a bottle of holy water suspended round her neck. Another wrote, "For a long time I endured my trials with patience, because one must suffer in order to obtain eternal life, but now my distress is greater than I can bear; my daughter's is not less than mine; I have persuaded her to die with me; we beseech God to pardon this crime, and we trust in Him."

*Belief in a future life; longing to rejoin lost friends.*—The daily necessities of our material existence, the satisfaction of the senses, the frivolity of the French character, and the complete indifference of the majority to the most solemn problem of humanity, account for the slight attention paid among us to what relates to God, eternity, and a future state. Perhaps it were more true to say that the sentiment of religion is repressed, rather than eradicated; still the general indifference on the subject shows that there is something radically wrong in our system of religious education. Twenty-two documents (eighteen men, four women) prove that the belief in another life is a consolation even for suicides. Some state that, being miserable here, they go in search of happiness in another world, to see if they shall be better off there. One writes, somewhat impiously, "Here I am, in full dress, my head well up, my conscience clear, ready to appear before the supreme tribunal." Others, in despair at the loss of a beloved person, go to rejoin them in eternity. One young man informs his family that he goes to find his mother, for whose loss he cannot be consoled. "Plunged in despair by the death of my child and beloved wife," writes another man, still quite young, "I kill myself to live with them in eternity." The letters of four females indicate as the motive, the desire of being reunited to those they loved.

*Wish for the prayers of the church; refusal.*—The idea of suicide is naturally incompatible with real religion, nevertheless, they are sometimes strangely associated, and present a distressing page in the study of that inexplicable mystery, the human heart. Thus, we have eleven persons (ten men, one woman), who probably during their lifetime rarely entered the doors of the Church, always opened to receive them, yet who desire to be admitted after their death, when the anathema has for ever closed her portals against them. The most frequent expressions are, that the writers die in the Catholic faith, that they desire to be buried with the customary ceremonies of the church, and that masses may be said for them. Occasionally it is evident that the suicide is desirous merely to save appearances, as in the following case: "You will do me a great service if you will go and inform the curate that I died from a stroke of apoplexy, so that I may receive the prayers of the church, and the manner of my decease remain unknown."

*Commend their souls to God.*—At the approach of death, and in the hour of trial, the feeling of religion is awakened and the name of God comes at once to the lips. Six documents (five men and one woman) attest that these were the last thoughts of so many suicides. The following are extracts from their letters: "I have just commended my soul to God, and said my prayers."—"In another hour my torments will be over; my last moments will be spent in prayer."—"I solicit God's forgiveness for my sin, may He have pity on his servant."—"I commend my soul to God in the name of our Saviour; may he receive it into grace; my affliction was beyond my strength."

5th Sub-section.—*Self-reproach at having yielded to seduction.*—There is a radical defect in the education and management of our women which calls for the earnest attention of moralists and governments.* Year after year thousands of illegitimate births, abortions, infanticides, and adulteries, reveal the breadth and profundity of the evil. A prey to continual attacks, the fall of these unfortunate creatures is too easily explained. Seduction is their deplorable heritage. We have nine letters on this head, and nothing can be more painful than the perusal of them. It is almost always the same sad tale: perjury, falsehood, and lying promises of marriage, are the beginning of their misfortunes. See what they tell us:—

* Feuchtersleben calls the female education of our times "the *partie honteuse* of the moderns."—Tr.

"After having promised to marry me, you have shamefully abandoned me. I forgive you, but I cannot survive the loss of my honour and your love." This letter finishes with the words, "I no longer see clearly."—"Your desertion and contempt are the causes of my death; still I would have lived if you had acknowledged our child."—"I commend my child to the care of the worthy ecclesiastic who has often consoled me. May misfortune overtake the seducer who ruined me: my spirit will haunt him everywhere." A poor girl relates, in touching language, the whole scheme of villany by which she fell, and the subsequent indifference and desertion of her seducer: being pregnant, she cannot survive her disgrace: she concludes—"God will punish the wretch who reduced me to this extremity." A poor woman, after being abandoned, writes to her daughter a letter, in which she explains to her all the misfortunes which await her, and enjoins her to follow the same course. The two were found together asphyxiated.

Although in this analysis of the last sentiments of suicides we have hitherto scrupulously confined ourselves to the 4595 official reports, yet we think the following statement, taken from the papers of the day, may be suitably introduced:—

"A young man, whose father holds an important official position, maintained for three years very intimate relations with a young widow, of restricted means. A few days ago he notified to his mistress that their *liaison* must terminate. The young woman made no complaint: that night was passed in watchfulness and tears; and on the following morning she sent for a porter, to whom she gave a letter and a small packet, with particular directions not to deliver them before the evening. She then shut herself in her room. It happened, however, that the porter having another job, which took him to the quarter indicated on the letter, delivered it before the appointed time. M. de M—— was at home, and upon reading the letter, which informed him that the unfortunate creature he had abandoned had terminated her sorrows by suicide, ran immediately to the commissary of police, and sent him to her abode. When the commissary arrived she still breathed, and the medical man who had accompanied him, recognising the symptoms which indicate poisoning by laudanum, at once commenced very vigorous treatment. In the meanwhile the commissary took note of the following letter: "Charles, you come not! You do not know, then, how much I suffer, and that my only wish was to see you for the last time. Do not despise me when you learn that I have voluntarily terminated my days. You know my character: it was not strong enough to enable me to bear up against the affliction of losing you. When you receive this letter I shall have ceased to live. I have not written this to alarm you, but to tell you once more that my last sigh was for you,—that I love you, and implore your forgiveness for my fatal resolve. I send you a ring plaited with my hair; wear it as a sign of pardon and remembrance." The letter concludes with instructions for her burial, to which she devotes the little property she dies possessed of.*

* We subjoin the following anecdote, taken from a recent number of "Galignani's Messenger:"—Tr.

"Yesterday the curiosity of the inhabitants of a house in the Rue St. Honore was attracted by seeing a canary-bird flying about the court-yard, which was observed to have a small strip of paper attached to its neck. The bird was at length caught, and the paper found to contain the words:—'Poor, ill, without employment, and without resource of any kind, I know not what will become of me; I am twenty years of age, and I cannot consent to lead a disreputable life. My mind is made up; all will be over this evening. The only friend I have in this world is this little bird, which I am now about to set at liberty. I beseech the person who finds it to take care of it, for it sings most sweetly, poor little creature. Thanks, thanks, beforehand.—Josephine.' As yet no clue to the writer of the billet has been discovered."

On summing up the various sentiments expressed in this chapter, we find that the first relate to man's social relations, manifested in his farewell to society. These adieus themselves follow in a hierarchal succession corresponding to the natural gradation of human affections, and are addressed successively to spouses, parents, children, lovers, mistresses, friends, acquaintances, and to the world at large. The majority of individuals in this category at the same time declare themselves the authors of their own death. More frequently they say nothing of the motives of their suicide, or, if they indicate them, attribute them to causes generally known.

A large number of letters finish with wishes, recommendations, and expressions of kindness and gratitude.

The sentiments placed in the second section relate principally to man's duties. The neglect of them torments the offenders. They acknowledge their fault, express regret at their unavailing attempts at reformation, punish themselves for their excesses, and are unwilling to dishonour their families.

In opposition to these last, there are some victims to an exaggerated sense of honour, which renders the slightest calumny, accusation, or suspicion, quite insupportable.

The sentiments expressed in the third section are those belonging to the family—love, friendship, and that general benevolence which we durst no longer style fraternity. The persons we have placed in this series regret the grief which their suicide will cause their parents or children, and the persons they love, and ask their pardon. They evince great solicitude for the welfare of their children, their wives or husbands, their parents, &c. &c.

Grief at parting from those they love is particularly felt by women, who are often inconsolable for their loss. A certain number alleviate the bitterness of separation by the hope of being remembered and wept for after their death. Others, on the contrary, are anxious that their end should be for ever concealed from all they knew, so that none may grieve over their untimely fate.

Forgetfulness of injuries, pardon for offences, kind regards for their fellow-creatures, are often shown by suicides in their last moments, and may be advantageously contrasted with the implacable hatred and malice frequently manifested in the wills of persons supposed to have died at peace with all the world.

The analysis of the fourth section comprises the religious sentiments. These often awaken forcibly at the approach of death, more particularly in women. In this resuscitation of devotional feeling, the idea of the One God is that which is most commonly presented to the mind; but in some, the religious sentiment goes beyond this, and induces them to solicit the prayers and ceremonies of the church in which they were brought up. We think it our duty to remark, that these documents prove the insufficiency of religious instruction among us, and show, without doubt, that the spirit is too much sacrificed to the letter.

Our fifth and last section is devoted to the sentiments expressed by the victims of seduction. The majority of these unfortunates forgive those who ruined them; but a few give way to recrimination. Our feelings are painfully affected at this review of the snares and dangers to which the weaker and gentler sex is exposed, and of which the terrible fruits are, bastardy, abortion, adultery, rape, prostitution, disgrace, and suicide.*

* We trust the learned author will forward to us the conclusion of this essay in time for our next number.

